# The PDZ Protein GIPC Regulates Trafficking of the LPA_1_ Receptor from APPL Signaling Endosomes and Attenuates the Cell’s Response to LPA

**DOI:** 10.1371/journal.pone.0049227

**Published:** 2012-11-08

**Authors:** Tal Varsano, Vanessa Taupin, Lixia Guo, Oscar Y. Baterina, Marilyn G. Farquhar

**Affiliations:** Department of Cellular and Molecular Medicine, University of California San Diego, La Jolla, California, United States of America; Hertie Institute for Clinical Brain Research and German Center for Neurodegenerative Diseases, Germany

## Abstract

Lysophosphatidic acid (LPA) mediates diverse cellular responses through the activation of at least six LPA receptors – LPA_1–6,_ but the interacting proteins and signaling pathways that mediate the specificity of these receptors are largely unknown. We noticed that LPA_1_ contains a PDZ binding motif (SVV) identical to that present in two other proteins that interact with the PDZ protein GIPC. GIPC is involved in endocytic trafficking of several receptors including TrkA, VEGFR2, lutropin and dopamine D2 receptors. Here we show that GIPC binds directly to the PDZ binding motif of LPA_1_ but not that of other LPA receptors. LPA_1_ colocalizes and coimmunoprecipitates with GIPC and its binding partner APPL, an activator of Akt signaling found on APPL signaling endosomes. GIPC depletion by siRNA disturbed trafficking of LPA_1_ to EEA1 early endosomes and promoted LPA_1_ mediated Akt signaling, cell proliferation, and cell motility. We propose that GIPC binds LPA_1_ and promotes its trafficking from APPL-containing signaling endosomes to EEA1 early endosomes and thus attenuates LPA-mediated Akt signaling from APPL endosomes.

## Introduction

Lysophosphatidic acid (LPA) mediates diverse biological effects including cell migration, differentiation, proliferation and survival [1], [2]. LPA induces these effects by binding to, and activating at least six different G protein coupled receptors (GPCRs), termed LPA_1_ through LPA_6_ [1]–[3], which are differentially expressed in different tissues and have distinct effects in animal models [1], [2]. These receptors are coupled to three classes of heterotrimeric G proteins, G_q/11_, G_i/o_ and G_12/13_, which mediate cellular responses to LPA [1], [2].

LPA receptors 1–3 are the most studied and share high sequence homology (∼55% overall sequence identity) except for their carboxy-terminus (CT) [3], [4]. LPA_1_ and LPA_2_ but not LPA_3_ contain the Class I PDZ binding motif sequence X-(S/T)-X-(V/I/L)-COOH (where X is any amino acid) at the extreme CT [3]. LPA_2_ CT, but not LPA_1_ or LPA_3_, interacts with the PDZ domain proteins NHERF2 and MAGI-3 which couple LPA_2_ to PLC-β3, RhoA and Erk signaling [3], demonstrating that the CT can couple LPA receptors to specific signaling pathways and thereby confer the specificity of the responses to each receptor [3], [4].

We noticed that LPA_1_ has a PDZ binding motif (SVV) identical to that present in two other proteins, semaphorin family member SemF and the melanosomal membrane protein GP75 [5], [6], which interact with the PDZ protein GIPC [7]. Like LPA_1_, GIPC plays a key role in cell motility as GIPC (a.k.a. Synectin) knock out mice have defects in endothelial cell migration and angiogenesis [8], [9]. We therefore wondered if GIPC might interact with the PDZ binding motif of LPA_1_ to regulate its activity.

GIPC (GAIP-interacting protein, C terminus) was originally identified based on its ability to bind to the RGS (regulator of G protein signaling) protein GAIP (RGS19), a GTPase activating protein (GAP) for heterotrimeric G proteins [7]. We subsequently found that GIPC binds to the TrkA nerve growth factor receptor [10]–[11] and is required for efficient endocytosis and trafficking of TrkA from peripheral (APPL) signaling endosomes to juxtanuclear (EEA1) endosomes [11]. GIPC accomplishes this in part by binding to the actin based molecular motor myosin VI (Myo6) [12] and in part by binding to APPL [11], [13], a Rab5 effector protein found on a subpopulation of peripheral endosomes. APPL is required for recruitment of GIPC to endosomes, and regulates key events in signal transduction from endosomes [14]–[16]. Additional studies demonstrated that GIPC also binds to the receptor tyrosine kinase VEGFR2 [17] as well as to G protein coupled receptors (GPCRs) such as the lutropin (hLHR) [18] and dopamine D2 (D2R) receptors [19] and promotes their endocytic trafficking. Previous studies of LPA_1_ trafficking indicate that LPA_1_ is taken up by endocytosis in clathrin coated pits, traffics through Rab5 endosomes, and recycles back to the cell surface [20]–[22]. Thus, we reasoned that interaction between GIPC and LPA_1_ might also affect trafficking of LPA_1_.

Here we show that GIPC directly binds to the PDZ binding motif of LPA_1,_ forms a complex with LPA_1_ and APPL, and promotes LPA_1_ trafficking from APPL signaling endosomes to early endosomes, resulting in downregulation of LPA_1_ induced Akt signaling and cell proliferation.

## Experimental Procedures

### Vectors

GIPC1 and APPL1 constructs were as previously described [10], [11]. GST-fusion proteins were cloned into the pGEX4T3 vector (GE Healthcare). LPA_1_ and LPA_2_ cDNAs cloned into pFLAG-CMV1 expression vector were obtained from Dr. Jerold Chun (Scripps Research Institute) [23] and subcloned into pIres-Puro3 vector (Clontech, Mountain View, CA).

### Antibodies

Rabbit anti-GIPC serum was affinity purified on GST-GIPC immobilized on PVDF membranes as described [11]. Rabbit anti-APPL serum was characterized previously [24]. Anti-MAP kinase (Erk1/2) mAb was purchased from Zymed Laboratories (San Francisco, CA), and anti-clathrin heavy chain (X22) mAb was from Affinity Bioreagents (Thermo Scientific, Rockford, IL). Rabbit antibodies against pERK (phospho-p44/p42) MAP kinase (Thr202/Tyr204), and pAkt (Ser473) were purchased from Cell Signaling Technology (Beverly, MA). Rabbit anti-FLAG and mouse anti-actin, anti-FLAG (M2), anti-PKBα/Akt, and anti-EEA1 IgG were obtained from Transduction Laboratories, BD Biosciences (San Diego, CA). Affinity purified mouse anti-HA (HA.11) IgG was from Covance (Berkeley, CA).

### Cell Culture and Transfection

HEK-293T cells were from Thermo Scientific (Rockford, IL), and HeLa cells were from the American Type Culture Collection (ATCC, CCL2). HEK-293T and HeLa cells were maintained in DMEM containing 10% FBS with 30 U/ml penicillin, 30 µg/ml streptomycin, 2 mM L-glutamine (GIBCO Invitrogen, Grand Island, NY). Clones stably expressing FLAG-tagged LPA1 receptor (HEK-LPA_1_) or controls (HEK-pIRES) were generated by transfecting pIres-Puro3- LPA_1_ or pIres-Puro3 empty vector, into cells using Lipofectamine 2000 (Invitrogen Corp., Carlsbad, CA) and selected by resistance to puromycin (2 µg/ml). HEK-293 and HeLa cells were transfected using Lipofectamine 2000 according to manufacturer’s instructions.

### Immunoprecipitation

Cells were lysed on ice for 30 min in lysis buffer (1% NP-40, 50 mM Tris, pH 8.0, 150 mM NaCl, 5 mM NaF, 2 mM sodium orthovanadate, and protease inhibitor cocktail (Sigma-Aldrich, St. Louis, MO). Insoluble material was removed by centrifugation (10,000×g for 30 min at 4°C), and the protein concentration of the supernatant was determined by Bradford assay (Bio-Rad Laboratories, Hercules, CA). Cell lysates (3–4 mg protein) were incubated at 4°C with mouse anti-FLAG IgG overnight followed by incubation with protein G-Sepharose beads (Sigma-Aldrich) for 1 h. Beads were then washed extensively with lysis buffer and resuspended in Laemmli sample buffer (50 mM Tris-HCl, pH 6.8, 2% SDS, 10% glycerol, 5% β-mercaptoethanol and 0.01% bromophenol blue) for SDS-PAGE.

### Immunoblotting

Proteins separated by SDS-PAGE were transferred to PVDF membranes (Millipore, Billerica, MA). After blocking with PBS containing 5% nonfat milk, membranes were incubated with primary antibodies at room temperature (1 h) or at 4°C (overnight), followed by incubation (1 h) at room temperature with goat anti-rabbit Alexa Fluor 680 F(ab’)_2_ (Molecular Probes) and goat anti-mouse IRDye 800 F(ab’)_2_ (Rockland). Infrared imaging with two-color detection and quantification of Western blots was performed according to the manufacturer’s protocols using the Odyssey Infrared Imaging System (LiCor Biosciences, Lincoln, NE).

### RNA Interference

Knockdown in HEK cells was achieved using a duplex siRNA targeting human GIPC1 (sense sequence 5-AGAGGUGGAAGUAUUCA-AGdT-dT) purchased from Dharmacon Inc., (Chicago, IL). A negative control siRNA (Silencer #1) was purchased from Ambion (Austin, TX). Transfection of HEK-293 cells was performed using Oligofectamine according to the manufacturer’s protocol (Invitrogen) with 50 nM siRNA, 0.8 µg/µl siRNA to lipid ratio, and a cell density of ∼ 100 cells/mm^2^ surface area.

### Protein Purification and In Vitro Binding Assays

GST, GST-GIPC, GST-mouse LPA_1_ tail (aa 311–364), GST-mouse LPA_2_ tail (aa 305–348) and mutants were expressed in *E. coli* and purified on glutathione Sepharose 4B (Amersham). For the in vitro binding assay 10 µg GST or GST fusion protein prebound to glutathione Sepharose beads were incubated with [^35^S]Met (GE Healthcare)-labeled GIPC-PDZ domain prepared using the TnT Quick Coupled Transcription/Translation System (Promega, Madison WI) in 300 µl binding buffer (50 mM Tris HCl, pH 7.4, 100 mM NaCl, 0.5%NP-40) overnight at 4°C. For experiments involving cell lysates, 3 µg GST or GST-GIPC were incubated with 500 µl cell lysate. Beads were sedimented and washed extensively in binding buffer and boiled in Laemmli sample buffer. Bead-bound proteins were separated by SDS-PAGE.

### Endocytosis Assay for LPA_1_

This assay was performed essentially as described previously [11]. HEK cells stably expressing LPA_1_ were grown on cover slips pre-coated with fibronectin (BD Biosciences, Bedford, MA). Cells were serum starved in DMEM at 37°C for 4 h, incubated on ice with anti-FLAG IgG (1∶1,000) for 0.5 h, washed with ice-cold PBS (3X), and shifted to fresh medium containing 1–10 µM LPA at 37°C for various times prior to fixation and processing for immunofluorescence.

### Immunofluorescence

HEK cells were fixed with 3% paraformaldehyde in 100 mM phosphate buffer, pH 7.4, for 30 min, permeabilized with 0.1% Triton X-100 in 1% BSA for 10 min, and incubated with primary antibodies for 1 h followed by goat anti-rabbit Alexa-594 and/or anti-mouse Alexa-488 F(ab')_2_ (Molecular Probes) for 1 h. Fluorescence images were taken with either an AxioImager M1 (Carl Zeiss, Thornwood, NY) equipped with a digital ORCA-ER camera (Hamamatsu), a PerkinElmer UltraView Vox Spinning Disk Confocal unit connected to an Olympus IX81 inverted microscope and a EMCCD camera (Hamamatsu), or an inverted Olympus FluoView 1000 confocal microscope equipped with a CH350 CCD camera (Hamamatsu). Images were processed with Adobe Photoshop 5.0 (Adobe Systems, Mountain View, CA). Fluorescence images of double-labeled samples were evaluated using the colocalization analysis features of the Volocity software (PerkinElmer, Waltham, MA).

### Deglycosylation Assay

Glycosylation assays (PNGase F treatments) were performed using the N-Glycanase-PLUS kit (ProZyme, San Leandro, CA) according to the manufacturer’s protocol. Briefly, HEK cells stably expressing FLAG-tagged LPA_1_ or empty vector were lysed in 0.1% SDS, 50 mM Tris HCl, pH 7.5, and 50 mM β-Mercapto-ethanol supplemented with protease inhibitors, and protein concentration was determined by the Bradford assay. Proteins (40 µg) were diluted in 45 µl of the above lysis buffer, and NP40 was added to a final concentration of 0.75%. 1 µl N-Glycanase-PLUS (Activity ≥10 U/ml) was added to half the samples, and the mixtures were incubated at 37°C for 3.5 h. Laemmli SDS sample buffer was added, proteins were resolved by SDS-PAGE, transferred to PVDF membranes, and analyzed by Western blotting using rabbit anti-FLAG IgG.

### Statistical Methods

Data in graphs are presented as the mean ± standard error of the mean (S.E.M) for *n* trials. Statistical analysis was carried out by Student's t-test, as appropriate, using 95% confidence limits. Specifics are detailed in the figure legends.

### Cell Migration Assay

Migration assays were performed as described by Klemke et. al. [25]. Briefly, Boyden chambers containing polycarbonate membranes (tissue culture-treated, 6.5 mm diameter, 10 µm thickness, 8 µm pores, Transwell®; Costar Corp., Cambridge, MA) were coated on both sides with human fibronectin for 2 h at 37°C. Cells were transfected with control or GIPC siRNA, and after 24 h they were incubated in serum free DMEM for an additional 24 h. 1×10^5^ cells in 100 µl serum free DMEM containing 1 mM sodium pyruvate and 0.25% fatty acid free BSA were added to the top of each well; the bottom of each well contained the same medium with or without 1 µM LPA. Cells were allowed to migrate for 3 h at 37°C and subsequently stained with crystal violet (Sigma). Cells that migrated to the bottom of the filter in each well were counted under the microscope to assess cell migration.

### Cell Proliferation Assay

Cell proliferation was assessed using a previously described crystal violet staining method [26]. Briefly, HEK cells stably expressing LPA_1_ or empty vector were transfected with control siRNA or GIPC siRNA in 12 well plates using lipofectamine 2000. 24 h after transfection cells were trypsinized, and 2×10^4^ cells were transferred to each well of a 96 well plate and cultured at 37°C. At specific time points (0–72 h) cells were fixed with 3.7% paraformaldehyde for 5 min, and stained with 0.05% crystal violet for 30 min. To determine cell numbers, the crystal violet in the wells was solubilized in methanol and absorbance (OD 540 nm) determined directly using a plate reader.

## Results

### GIPC Specifically Interacts with the PDZ Binding Motif of LPA_1_

**Figure 1:**
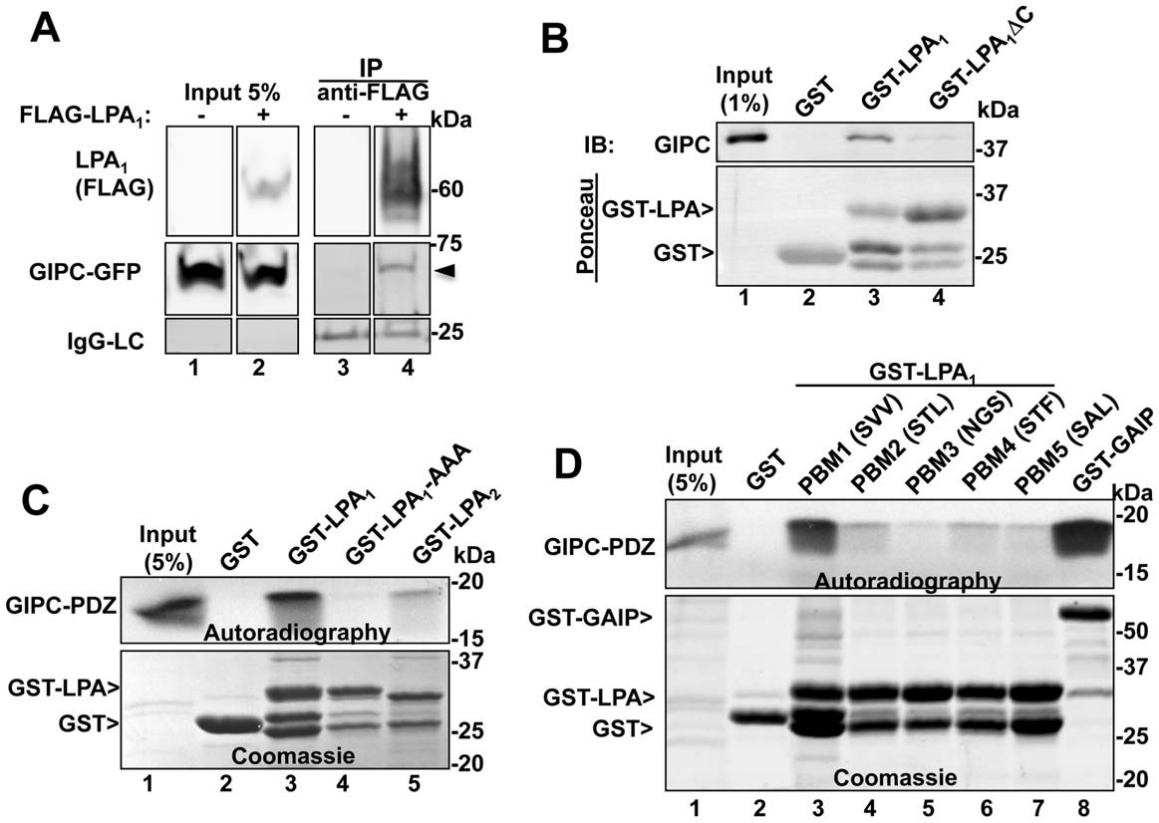
GIPC directly interacts with the C-terminal PDZ binding motif of LPA_1_ but not with other LPA receptors. A, Endogenous GIPC and GIPC-GFP co-immunoprecipitate with FLAG- LPA_1_ from HEK cells expressing FLAG- LPA_1_ (arrowhead, lane 4) but not control HEK cells (lane 3). C-terminally tagged GIPC-GFP and N-terminally tagged FLAG-LPA_1_ were transiently coexpressed in HEK293 cells, and immunoprecipitation was carried out on cell lysates with mouse anti-FLAG IgG followed by immunoblotting with mouse anti-FLAG (LPA_1_) and rabbit anti-GIPC IgG. Lanes were cropped from a single exposure of a continuous membrane. The lower panel shows the amount of IgG light-chain (IgG-LC) in each IP. Lanes 1–2*:* Input showing the amounts of LPA_1_ and GIPC present in the lysates used for the IP. B, *Upper panel*: GIPC binds GST-LPA_1_ (GST fused to the cytoplasmic tail of mouse LPA_1_ (aa 311–364), lane 3) but not to GST alone (lane 2) or GST-LPA_1_ΔC (lacking the last three C-terminal amino acids, lane 4). Immobilized recombinant GST, GST-LPA_1_ and GST- LPA_1_ΔC were incubated 4–15 h with lysates from HEK293 cells transiently transfected with FLAG-GIPC. Proteins bound to immobilized fusion proteins were eluted with 2X sample buffer for SDS-PAGE and immunoblotted with anti-GIPC IgG. Lane 1: input, showing the amount of GIPC in 1% of the lysate used for the assay. *Lower panel* : Ponceau staining demonstrating the amount of GST proteins used in each assay. C, *Upper panel*: Autoradiography showing that in vitro translated, [^35^S]GIPC PDZ domain binds to GST-LPA_1_ (lane 3) but not to GST alone (lane 2), GST- LPA_1_AAA (last three amino acids mutated to alanine, lane 4), or GST-LPA_2_ (lane 5). GST fusion proteins were immobilized on glutathione-agarose beads as in “B” and incubated with in vitro translated [^35^S]Met-labeled, GIPC PDZ domain (aa 125–225). Bound proteins were separated by SDS-PAGE and detected by autoradiography. Lane 1: 5% of the in vitro translated protein. *Lower panel* : Coomassie Blue staining showing the GST proteins used for the assay. D, *Upper panel:* Autoradiography showing that in vitro translated, [^35^S] GIPC-PDZ interacts with the C-terminal PDZ binding motif of LPA_1_ (SVV, lane 3) and with GST-GAIP (lane 8, used as a positive control [7] but shows little or no interaction with GST alone (lane 2) or GST-LPA_1_ mutants in which the three C-terminal amino acids were modified to those of LPA_2_ (STL, lane 4), LPA_3_ (NGS, lane 5), LPA_4_ (STF, lane 6) or LPA_5_ (SAL, lane 7). Immobilized GST fusion proteins were incubated with in vitro translated [^35^S]Met-labeled GIPC PDZ and analyzed as in C. *Lower panel*: Coomassie Blue staining showing the amounts of GST proteins used.

To determine if GIPC can interact with LPA_1_ we transiently co-expressed GIPC-GFP and N-terminally tagged FLAG- LPA_1_ in HEK293 cells and immunoprecipitated LPA1 with anti-FLAG IgG. We found that GIPC-GFP co-immunoprecipitated with FLAG-LPA_1_ (Fig. 1A), suggesting that GIPC and LPA_1_ are present in the same protein complexes.

To determine if GIPC interacts with the PDZ binding motif of LPA_1_ we carried out GST pull-down assays with GST-LPA_1_ (aa 311–364) on cell lysates from HEK293 cells transiently transfected with FLAG-GIPC. We found that GIPC bound to GST-LPA_1_ (Fig. 1B, lane 3), but did not bind to GST-LPA_1_ΔC, lacking the PDZ binding motif (-SVV) (Fig. 1B, lane 4). To find out if the interaction between GIPC and LPA_1_ is direct and whether the PDZ domain of GIPC is sufficient for the interaction we performed pull down assays using GST-fusion proteins and [^35^S] Met-labeled, in vitro translated, GIPC-PDZ (aa 125–225). GIPC-PDZ bound to GST-LPA_1_ (Fig. 1C, lane 3) but not to GST-LPA_1_-AAA, a mutated version of GST- LPA_1_ in which the last three amino acids were mutated to alanine (Fig. 1C, lane 4). Interaction with the cytoplasmic tail of LPA_2_ was much weaker (Fig. 1C, lane 5) even though it also has a class-I PDZ binding motif. To verify the specificity of GIPC’s interaction with the PDZ binding motif of LPA_1_ we mutated the last three amino acids of LPA_1_ cytoplasmic tail (-SVV) to resemble the C-terminal sequence of LPA receptor subtypes 2 (-STL), 3 (-NGS), 4 (-STF) and 5 (-SAL). In vitro translated GIPC-PDZ bound to the PDZ binding motif of LPA_1_ whereas interactions with other PDZ binding motifs were much weaker (Fig. 1D), suggesting that GIPC interacts specifically with LPA_1_ and can distinguish the PDZ binding motif of LPA_1_ from closely related PDZ binding motifs of other members of the LPA receptor family. Taken together these results demonstrate that GIPC directly binds to the PDZ binding motif of LPA_1_, that this interaction is specific for LPA_1_, and that it is mediated via the PDZ domain of GIPC and the C-terminal PDZ binding motif of LPA_1_.

### LPA_1_ and GIPC Traffic Together to APPL Endosomes

**Figure 2:**
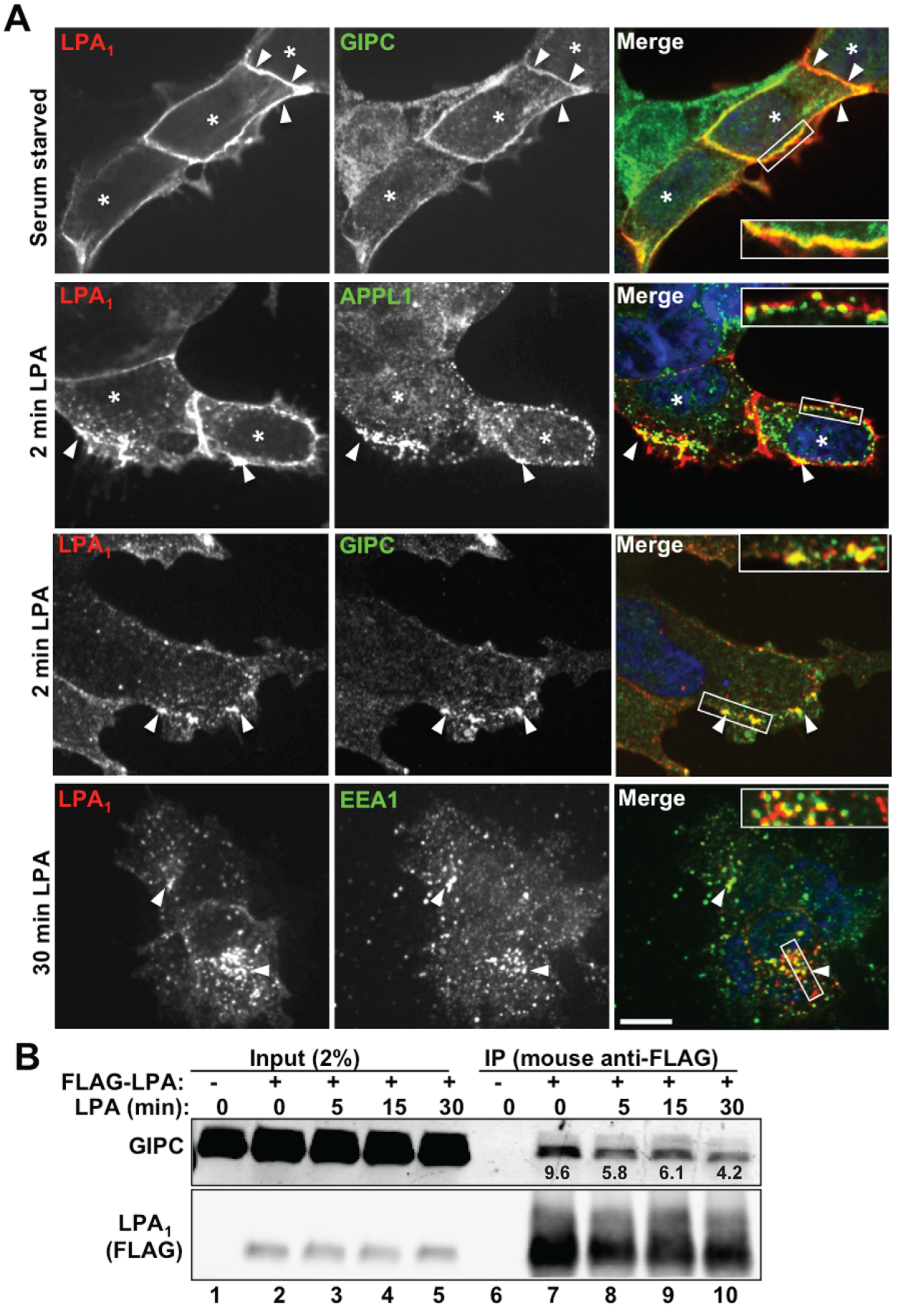
Trafficking of LPA_1_ and GIPC to Endosomes. A, *Upper panel*: In serum starved cells stably expressing LPA_1_ (asterisks), GIPC is concentrated at the plasma membrane (arrowheads) where it colocalizes with LPA_1_. *Middle panels* : 2 min following stimulation with LPA, LPA_1_ colocalizes with APPL and GIPC (arrowheads) in endocytic vesicles at the cell periphery. *Lower panel* : 30 min following stimulation, LPA_1_ colocalizes with EEA1 in early endosomes concentrated in the juxtanuclear region (arrowheads). Boxed regions are enlarged (2.2×) in the insets. Bar = 10 µm. HEK- LPA_1_ cells were serum starved for 4 h, incubated on ice with mouse anti-FLAG IgG to label FLAG-LPA_1_ at the cell surface, washed in PBS and shifted to fresh medium containing LPA (10 µM) 2 or 5 min before fixation. Cells were processed for immunofluorescence using affinity purified rabbit anti-GIPC, anti-APPL1 or anti-EEA1 IgG, followed by goat anti-rabbit Alexa-488 and goat-anti-mouse Alexa-594 F(ab’)_2_ (the latter to detect FLAG-LPA_1_). Images were taken with a PerkinElmer UltraView Vox Spinning Disk Confocal unit connected to an Olympus IX81 inverted microscope and a EMCCD camera (Hamamatsu) using a 60X oil immersion lens (1.42 NA). B, GIPC co-immunoprecipitates with FLAG-LPA_1_ from serum-starved cells (lane 7) at all time points after LPA stimulation, but the interaction gradually decreases after LPA stimulation (lanes 8–10). The relative abundance of GIPC that coprecipitated with FLAG-LPA_1_ is indicated beneath each band. As expected, both LPA_1_ and GIPC are absent from immunoprecipitates of cells transiently transfected with empty vector instead of FLAG- LPA_1_ (lane 6, vector control). HEK293 cells were transiently co-transfected with full-length GIPC and FLAG-LPA_1_ (lanes 2–5 and 7–10) or GIPC alone (lanes 1 and 6). Cells were serum starved overnight (lanes 1, 2, 6 and 7) or starved and stimulated with 10 µM LPA for 5 (lanes 3 and 8), 15 (lanes 4 and 9) or 30 min (lanes 5 and 10) before lysis. IP was carried out on cell lysates using mouse anti-FLAG IgG and immunoblotted as is Fig. 1A. The abundance of GIPC and LPA_1_ in each immunoprecipitation reaction was quantified using the LICOR imaging system, and the GIPC abundance relative to LPA_1_ was calculated for each reaction. Similar results were obtained in 2 additional experiments. Input (lanes 1–5): Lysates (2%) are shown to verify comparable expression levels.

**Figure 3:**
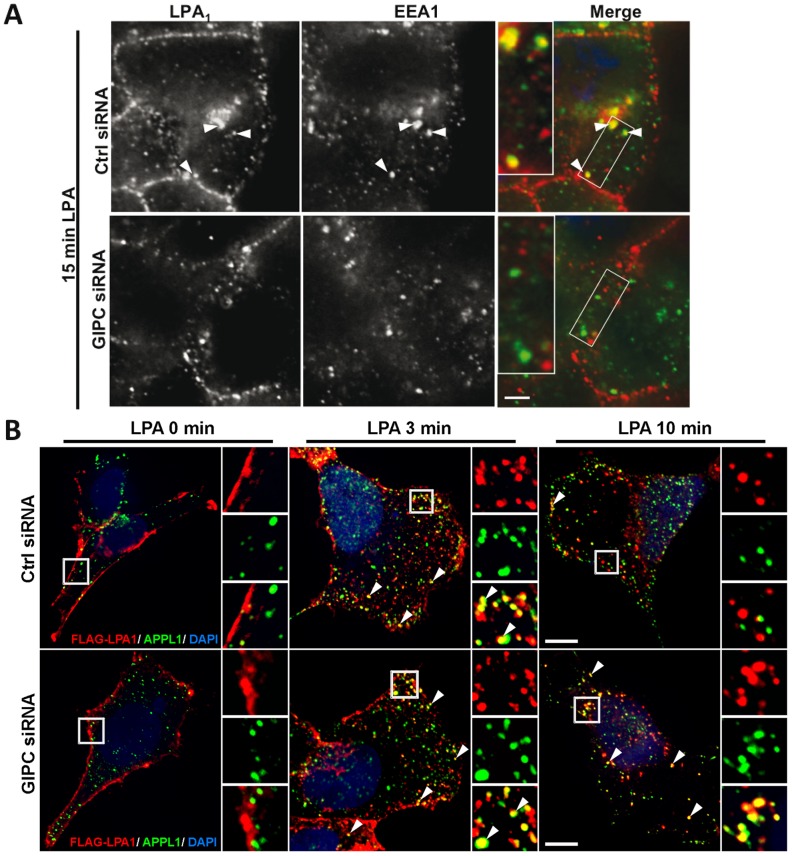
GIPC depletion delays trafficking of LPA_1_ from APPL1 to early EEA1 endosomes. A, GIPC depletion inhibits internalization of LPA_1_ and its trafficking to early endosomes after stimulation with LPA. *Upper panel:* In HEK-LPA_1_ cells transfected with control siRNA and stimulated with LPA for 15 min, LPA_1_ is found in cytoplasmic vesicles where it colocalizes with EEA1 (arrowheads). *Lower Panel:* In cells transfected with GIPC siRNA fewer vesicles containing LPA_1_ are present 15 min after LPA stimulation, and less colocalization is seen between LPA_1_ and EEA1 (compare yellow in right panels). Boxed regions are enlarged (2.2×) in the insets. Images were acquired with a Zeiss AxioImager M1 microscope, and overlap in staining between LPA_1_ and EEA1 was evaluated using Volocity software. Statistical significance (p value) was determined by t-test. B, Trafficking of LPA_1_ is delayed in APPL1 endosomes after depletion of GIPC. *Left panel:* In both GIPC-depleted (GIPC siRNA) and controls (Ctrl siRNA), LPA_1_ is localized along the plasma membrane after serum starvation (0 min) whereas APPL1 is found in peripheral cytoplasmic vesicles. *Middle Panel:* In both GIPC depleted and control cells stimulated with LPA for 3 min, LPA_1_ colocalizes with APPL1 in cytoplasmic vesicles (arrowheads). *Right Panel:* In controls stimulated with LPA for 10 min, very few LPA_1_ receptors remain in APPL endosomes (yellow, arrowhead) whereas in GIPC-depleted cells the majority of the receptors are retained in APPL endosomes (yellow, arrowhead). Boxed regions are enlarged (3×) in the insets. HEK-LPA_1_ cells grown on coverslips were transfected with GIPC or control siRNA. 72 h after transfection cells were serum starved for 4–6 h and subsequently incubated on ice with rabbit (A) or mouse (C) anti-FLAG IgG, shifted to fresh medium containing LPA for the indicated times, then fixed and processed for immunofluorescence using mouse anti-EEA1 IgG (A) or rabbit-anti-APPL1 IgG (C) as in Fig. 2A. Images in “A” were acquired with a Zeiss AxioImage M1 microscope, and those in “C” were acquired with an Ultra View Vox Spinning Disk Confocal. Bar = 10 µm.

We have previously shown [11] that GIPC binds to the receptor tyrosine kinase TrkA and regulates its trafficking and signaling through interaction with APPL, a Rab5 effector that serves as a marker for APPL signaling endosomes [14]–[16]. To investigate if GIPC similarly regulates trafficking and signaling of LPA_1_ we prepared HEK293 cell lines stably expressing FLAG- LPA_1_ (HEK-LPA_1_) or empty vector (HEK-pIRES) (Fig. S1). We chose HEK293 cells because they were previously shown to express LPA_1_ but not LPA_2_ or LPA_3_, [27], and therefore any response to LPA observed is likely to be via activation of LPA_1_ and its downstream signaling network. First we followed the trafficking of LPA_1_ and its association with GIPC and APPL in these cells. In serum starved HEK-LPA_1_ cells stably expressing LPA_1_, LPA_1_ colocalized with GIPC along the PM (Figs. 2A, and S3 upper panel). Similar results were also obtained in HeLa cells transiently expressing LPA_1_ (Fig. S2). By 2–5 min after stimulation with LPA, LPA_1_ had been partially internalized and accumulated on peripheral vesicles located just beneath the plasma membrane that colocalize with both GIPC and APPL (Fig. 2A, middle panels and S4 upper panel). Beginning at 15 min (see Figs. 3A and S4) and especially by 30 min after ligand stimulation (Figs. 2A and S4, lower panels) LPA_1_ colocalized with the early endosome marker EEA1 in the juxtanuclear region and no longer colocalized with either GIPC (Fig. S3) or APPL (Fig. S4). Thus our results suggest that, like TrkA [11], after agonist stimulation LPA_1_ is internalized and passes first through APPL endosomes located at the cell periphery and then to EEA1 early endosomes located in the juxtanuclear region.

To determine if LPA_1_ and GIPC are internalized via clathrin mediated endocytosis we performed double labeling for clathrin and GIPC or LPA_1_ (Fig. S5). We found that 2–3 minutes following addition of LPA, both LPA_1_ and GIPC colocalized with clathrin in punctate structures at or just beneath the plasma membrane indicating that following LPA stimulation, GIPC and LPA_1_ are internalized into clathrin coated pits which pinch off the plasma membrane to form clathrin coated vesicles.

To find out if ligand stimulation affects the association between LPA_1_ and GIPC we immunoprecipitated LPA_1_ from HEK293 cells transiently expressing FLAG-LPA_1_ before and after stimulation with LPA (5–30 min). GIPC co-immunoprecipitated with LPA_1_ at all time points, but the amount of GIPC that co-immunoprecipitated with LPA_1_ gradually declined after ligand stimulation (Fig. 2B). Collectively the immunofluorescence and biochemical results suggest that, as for TrkA [11], GIPC associates with LPA_1_ at the plasma membrane, GIPC and LPA_1_ travel together to APPL endosomes (2–5 min), and they dissociate from one another before LPA_1_ reaches early (EEA1) endosomes (30 min).

### GIPC Depletion Disrupts LPA_1_ Trafficking

Next we investigated the effects of GIPC depletion on LPA_1_ trafficking at 0 and 15 min after LPA stimulation. In serum starved cells LPA_1_ was present largely at the plasma membrane in both GIPC-depleted cells and controls (not shown). At 15 min after addition of LPA, in controls LPA_1_ appeared both at the PM and in vesicles inside the cell where it partially colocalized with the early endosome marker EEA1 (Fig. 3A, upper panel). By contrast in GIPC-depleted cells fewer vesicles with LPA_1_ were seen in the cytoplasm, and colocalization between LPA_1_ and EEA1 was markedly reduced (Fig. 3A, middle panel). Quantification of the overlap between LPA_1_ and EEA1 (Fig. 3B) using Volocity software revealed a 32% decrease in the average overlap coefficient (OC) in GIPC depleted cells (OC = 0.45) compared to controls (OC = 0.66). The decreased localization of LPA_1_ in EEA1 early endosomes at 15 min after LPA addition suggests that in GIPC depleted cells there is a delay in trafficking of LPA_1_ from the plasma membrane or peripheral vesicles to early endosomes.

To test if following GIPC depletion, LPA_1_ accumulates in peripheral (APPL) signaling endosomes we carried out double labeling for LPA_1_ and APPL1 0–10 min after LPA stimulation (Fig. 3C). We found that in control cells, colocalization between APPL1 and LPA_1_ in APPL endosomes peaked at 3 min and was barely detected at 10 min after LPA stimulation. In contrast, in GIPC depleted cells, colocalization between APPL1 and LPA_1_ increased 3 min after LPA stimulation but remained high even after 10 min. Taken together, these results suggest that GIPC promotes trafficking of LPA_1_ from peripheral APPL signaling endosomes to early endosomes after internalization of the receptor from the plasma membrane.

### GIPC Depletion Enhances LPA_1_ Signaling

**Figure 4:**
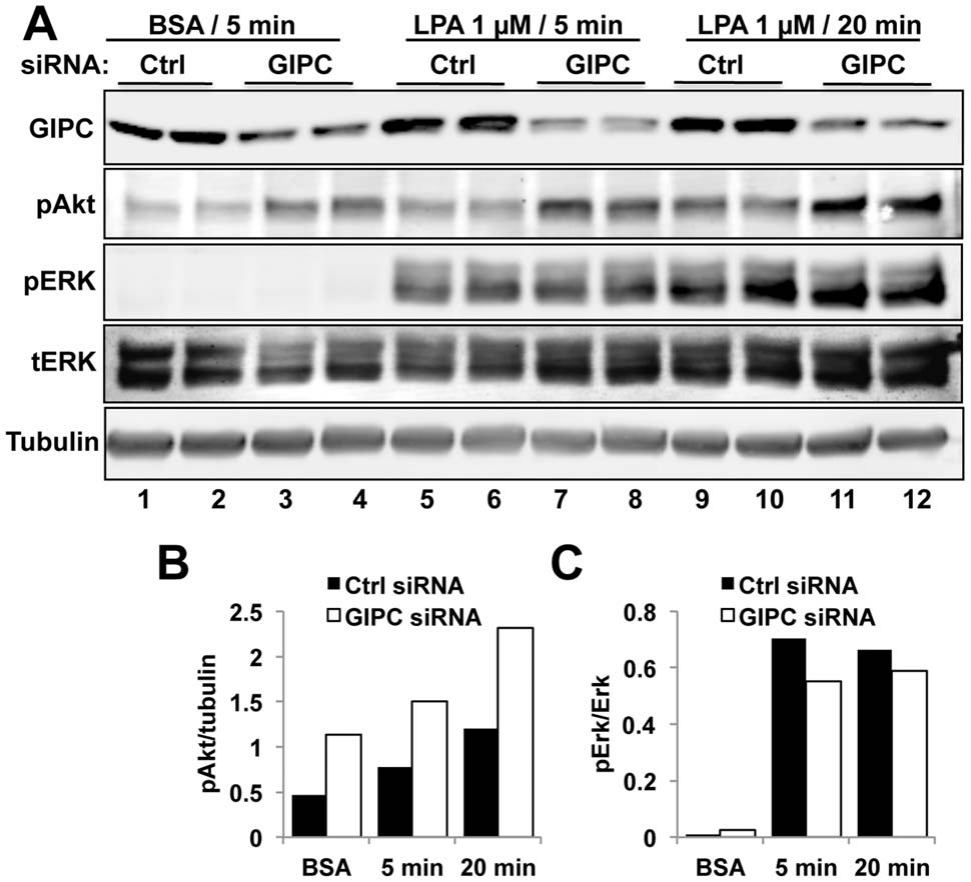
Akt phosphorylation is increased in GIPC depleted HEK-LPA cells. A, In HEK- LPA_1_ cells transfected with control siRNA both Akt and ERK1/2 activation are enhanced after stimulation with LPA for 5 (lanes 5–6) or 20 min (lanes 9–10). GIPC depletion (GIPC siRNA) enhanced Akt phosphorylation (pAkt) at 5 min (lanes 7–8) and 20 min (lanes 11–12) after LPA stimulation. GIPC depletion also enhanced Akt signaling in the absence of ligand (lanes 1–4), possibly due to enhanced basal activity of LPA_1_. Erk phosphorylation (pERK) was not affected by GIPC depletion. HEK-LPA_1_ cells were transfected with control or GIPC siRNA, serum starved overnight, stimulated with 1 µM LPA or incubated with BSA alone for 5 or 20 min, lysed in RIPA buffer and analyzed by immunoblotting using phospho-Erk (pErk), total Erk (tErk), phospho-Akt (pAkt) and α-tubulin IgG. Each treatment was done in duplicate. α-tubulin was used as a loading control. In cells transfected with GIPC siRNA (lanes 3–4, 7–8, 11–12), GIPC expression is reduced 70–80% in cells transfected with control siRNA (Ctrl, lanes 1–2, 5–6, 9–10). B–C, Densitometric analysis of the immunoblots in A demonstrating that GIPC depletion (siRNA) leads to a two-fold increase in Akt phosphorylation (B) at both 5 and 20 min after LPA stimulation (B, P<0.05) but does not significantly affect Erk phosphorylation (C) compared to controls (Ctrl siRNA).

**Figure 5:**
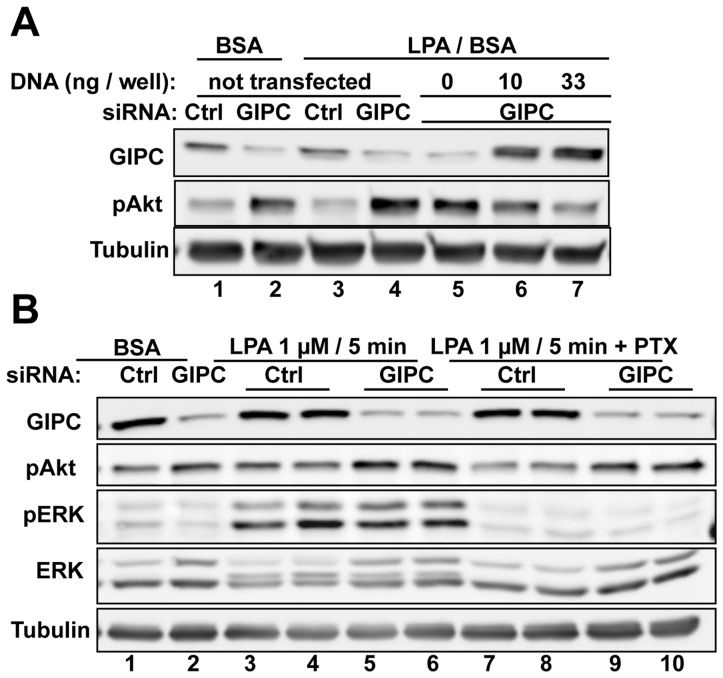
Enhancement of Akt activation following GIPC depletion is reversed by GIPC expression and is independent of Gαi signaling. A, GIPC depleted HEK-LPA_1_ cells show reduced Akt phosphorylation after transfection of siRNA resistant GIPC DNA (lanes 6–7, middle panel) verifying that GIPC is responsible for the enhanced Akt phosphorylation seen after GIPC depletion. HEK-LPA_1_ cells were transfected with GIPC or control siRNA, and 12 h later they were transfected with siRNA-resistant GIPC DNA (0, 10, or 33 ng). After 24 h cells were serum starved overnight, stimulated with 1 µM LPA for 5 min, and cell lysates were immunoblotted for GIPC, pAkt and tubulin (used as loading control). B, Activation of Gαi is not required for the enhanced Akt phosphorylation seen after GIPC depletion. In GIPC-depleted cells PTX treatment (lanes 7–10) prevented LPA induced Erk phosphorylation (pErk) (which is Gαi dependent) but did not affect Akt phosphorylation (pAkt) compared with controls (lanes 3–6). 36 h after siRNA transfection, HEK-LPA_1_ cells were cultured for another 12 h in serum-free media in the presence or absence of PTX and then stimulated for 5 min with 1 µM LPA (in 0.1% BSA, lanes 3–10) or incubated in BSA alone (0.1%) for 5 min (lanes 1–2).

To investigate the effects of GIPC depletion on LPA_1_ signaling we stimulated HEK-LPA_1_ cells with LPA and assessed activation (phosphorylation) of Erk and Akt–two signaling pathways that mediate cell survival, proliferation and motility. We found that at both 5 and 20 min after LPA stimulation GIPC depletion (∼70%) enhanced Akt activation by ∼2-fold (Fig. 4A and B) but had no effect on pErk levels (Fig. 4A and C). Similar findings were obtained using different clones of HEK-LPA_1_ cells. Transfection of siRNA resistant GIPC into GIPC depleted cells reversed the effect of GIPC siRNA on Akt phosphorylation in a dose dependent manner (Fig. 5A, lanes 5–7) verifying that the effects of GIPC expression on Akt phosphorylation are not due to off target effects of the siRNA.

We showed previously that GIPC recruits GAIP (RGS19), a GAP for Gαi proteins [28], and inhibits Gi signaling [10]. To determine if Gαi activity is required for LPA_1_ mediated Akt phosphorylation we pre-treated GIPC depleted and control cells with pertussis toxin (PTX), an inhibitor of Gαi/GPCR coupling, before LPA stimulation. PTX abolished LPA induced Erk activation but did not affect activation of Akt (Fig. 5B). Notably, PTX did not inhibit the increased Akt phosphorylation seen in GIPC depleted cells (Fig. 5B), indicating that the effect of GIPC on Akt phosphorylation is most likely not mediated through Gαi subunits.

### APPL is Present in LPA_1_ Complexes and APPL Depletion Inhibits Akt Activation

**Figure 6:**
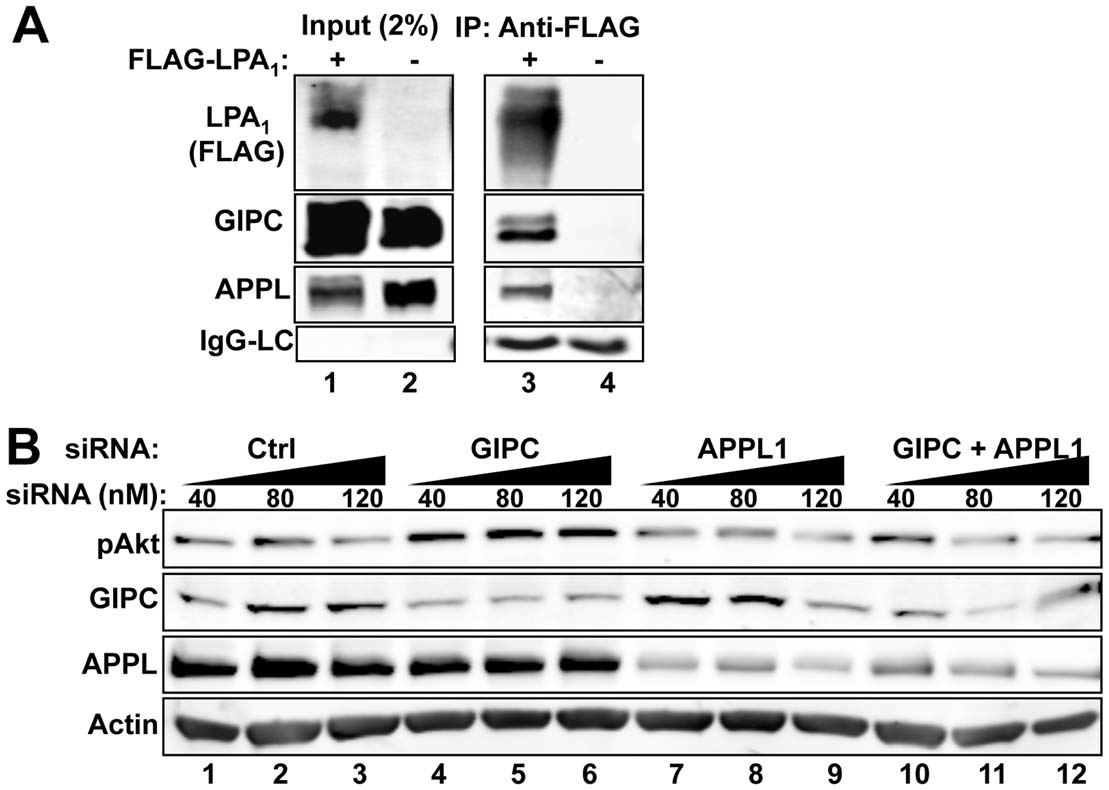
APPL interacts with LPA_1_ and affects LPA_1_ mediated Akt signaling. A, APPL and GIPC co-immunoprecipitate with FLAG- LPA_1_ (lane 3). HEK cells were co-transfected with HA-APPL and GIPC together with FLAG-LPA_1_ (lanes 1 and 3) or empty vector (lanes 2 and 4), and cultured in the presence of 10% PBS for 48 h before lysis. IP was carried out as in Fig 1A. Aliquots of cell lysates (input, 2%) were loaded to verify comparable expression levels. B, APPL depletion inhibits Akt activation in HEK-LPA_1_ cells stimulated with LPA. Depletion of GIPC (lanes 4–6) leads to increased Akt signaling (top panel) compared with controls (lanes 1–3). In contrast, depletion of APPL1 alone (lanes 7–9) or double knockdown of GIPC and APPL (lanes 10–12) results in reduced Akt signaling. HEK-LPA_1_ cells were transfected with increasing amounts of control (lanes 1–3), GIPC (lanes 4–6) or APPL siRNA (lanes 7–9) or GIPC and APPL siRNA combined (lanes 10–12). Cells were serum starved overnight, stimulated with 5 µM LPA for 15 min, lysed and analyzed by immunoblotting as in Fig. 3D.

APPL directly binds GIPC as well as the TrkA receptor [11], [13] and promotes Akt signaling and cell survival [14]. To determine whether LPA_1_ forms a complex with APPL and GIPC we immunoprecipitated FLAG-LPA_1_ from HEK-LPA_1_ cells at steady-state (10% FBS) and immunoblotted for APPL and GIPC. We found that APPL and GIPC co-immunoprecipitated with LPA_1_ (Fig. 6A, lane 3), indicating that LPA_1_ is present in the same protein complexes as GIPC and APPL.

To determine if APPL is required for enhancing Akt phosphorylation following GIPC depletion we treated HEK- LPA_1_ cells with control siRNA (Fig. 6B, lanes 1–3), GIPC siRNA alone (Fig. 6B, lanes 4–6), APPL siRNA alone (Fig. 6B, lanes 7–9) or both GIPC and APPL siRNA (Fig. 6B, lanes 10–12) and stimulated the cells with LPA. Depletion of GIPC led to enhanced Akt signaling as before, whereas depletion of APPL or double knockdown of GIPC and APPL reduced Akt signaling. The reversal of Akt enhancement in the double knockdown suggests that APPL is required for the enhancement of Akt signaling. Taken together, these results suggest that following ligand stimulation APPL associates with LPA_1_ protein complexes and mediates Akt activation downstream of LPA_1_.

### GIPC Depletion Promotes LPA_1_ Mediated Cell Proliferation and Cell Motility

**Figure 7:**
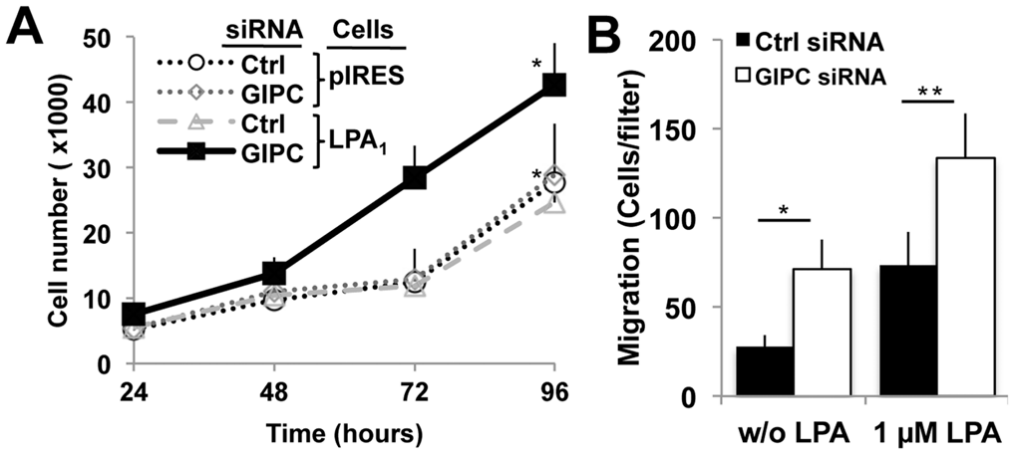
Depletion of GIPC in cells overexpressing LPA_1_ promotes cell proliferation and cell motility. A, GIPC-depleted HEK-LPA_1_ cells (solid line) grow faster than those transfected with control (Ctrl) siRNA (_*_, p<0.05). In contrast, GIPC depletion had little effect on the growth rate of HEK-pIRES cells which do not overexpress LPA_1_. HEK-LPA_1_ and HEK-pIRES cells were transfected with GIPC (siRNA) or control siRNA (Ctrl) and transferred to 96 well plates 24 h post-transfection. Cells were cultured for up to 72 h in medium containing 10% serum without puromycin. Cell number was determined as described in “Material and Methods”. Data are the mean ± s.e.m (n = 16 wells). B, Depletion of GIPC promotes HEK-LPA_1_ cell motility. Serum starved HEK-LPA_1_ cells were transfected with either GIPC (white bars) or control (black bars) siRNAs and allowed to migrate for 3 h on fibronectin-coated filters. LPA (1 µM) was added to the bottom chambers in half the wells. The number of migrating cells was determined by counting cells on the underside of the filters as described in “Material and Methods”. Each bar represents the mean ± s.e.m. of triplicate wells in three independent experiments.

Next we investigated if GIPC depletion can affect cell growth in the presence of LPA. GIPC was depleted from HEK-LPA_1_ cells, and the growth of GIPC depleted vs control HEK- LPA_1_ cells was followed for 96 h after siRNA transfection. GIPC depletion resulted in a 72% increase in the number of cells per well (43,000+/−6,000 vs 25,000+/−8,000 cells/well) (Fig. 7A). GIPC depletion did not significantly affect growth of HEK-pIres controls that do not express FLAG- LPA_1_ (Fig. 7A), indicating that enhancement of cell growth is mediated through LPA_1_. In addition, the number of HEK-LPA_1_ cells that incorporated BrdU was increased from 18% in controls to 23% in GIPC depleted cells (data not shown), suggesting a slightly faster cell cycle. These results are consistent with a role for GIPC in down-regulating LPA_1_ mediated cell growth or cell survival.

Because LPA_1_ is also known to trigger cell motility we next examined the effect of GIPC depletion on cell migration by analyzing movement of HEK-LPA_1_ cells across a porous membrane in a Boyden chamber in the presence of concentration gradient of LPA [25]. GIPC depletion enhanced motility of HEK-LPA_1_ cells in that increased numbers of cells migrated across the membrane both in the presence and absence of a concentration gradient (Fig. 7B). These results demonstrate that GIPC inhibits cell motility in cells expressing LPA_1_. Previously, LPA_1_ was shown to possess intrinsic basal activity even in the absence of ligand binding [29]. Thus the inhibitory effect of GIPC on cell motility in the absence of ligand is most likely due to inhibition of the basal activity of LPA_1_.

**Figure 8:**
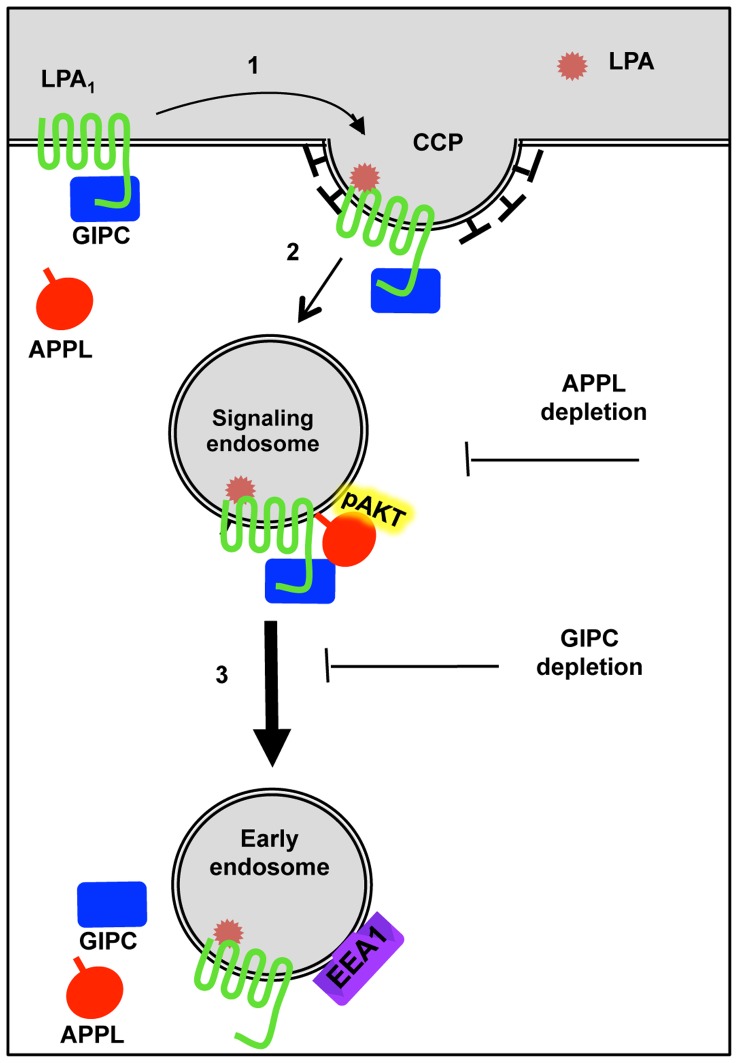
Working model depicting LPA_1_ endocytic trafficking and signaling and its interactions with GIPC and APPL. In the absence of ligand LPA_1_ is found at the plasma membrane in a complex with GIPC. When LPA is added, LPA_1_ and GIPC move into clathrin-coated pits (1). Clathrin-coated vesicles containing LPA_1_-GIPC complexes pinch off the cell membrane and uncoat and APPL is recruited (2). APPL binds pAkt to form peripheral signaling endosomes. GIPC, by binding to APPL and the motor protein myosin VI, facilitates movement of these endosomes to the juxtanuclear region (3). In juxtanuclear early endosomes, GIPC and APPL are released into the cytoplasm thus terminating APPL-pAkt signaling. Depletion of GIPC inhibits LPA_1_ trafficking to EEA1 endosomes and prolongs LPA_1_ signaling from APPL endosomes whereas depletion of APPL inhibits Akt signaling.

Based on our results we propose a working model (Fig. 8) in which GIPC associates with LPA_1_ at the PM in a ligand independent manner, and following ligand stimulation the receptor and GIPC are internalized in clathrin-coated vesicles and associate with APPL which activates Akt signaling. Subsequently, GIPC promotes LPA_1_ trafficking to early (EEA1) endosomes and thus terminates APPL/Akt signaling. Depletion of GIPC delays trafficking of LPA_1_, prolongs its stay in APPL signaling endosomes, and enhances Akt signaling leading to increased cell motility and cell proliferation.

## Discussion

We demonstrate here that GIPC binds LPA_1_ and that binding is direct and is mediated through the PDZ domain of GIPC and the C-terminal PDZ binding motif of LPA_1_. siRNA depletion of GIPC delayed trafficking of LPA_1_ to early endosomes and resulted in enhanced LPA_1_-mediated Akt signaling and enhanced cell proliferation and cell motility. APPL, a marker APPL/GIPC signaling endosomes, was present in LPA_1_ complexes and necessary for LPA_1_ mediated Akt signaling. Taken together, these results support a model in which GIPC promotes trafficking of LPA_1_ from APPL signaling endosomes to early (EEA1) endosomes thus attenuating LPA_1_ mediated signaling and cellular responses (see Figure 8).

Both LPA_1_ and GIPC have been implicated in cell migration [8], [17], [30]–[32], neuronal cell activity [33], [34] and cell proliferation [9], [35], [36]. GIPC has been shown to inhibit endothelial cell migration through interaction with Endoglin [37] or syndecan-4 [38], but it promotes migration of primary arterial endothelial cells [8]. LPA_1_ has been shown to promote migration and proliferation of many cell types [30]–[32], [35]. Our results showing that GIPC binds LPA_1_ and regulates its activity suggest a novel mechanism by which GIPC affects cell migration. We also observed an apparent increase in cell proliferation following GIPC depletion in cells expressing LPA_1_. The effects of LPA_1_ on cell proliferation are most likely indirect and are believed to reflect a combination of the secondary release of growth factors following initial LPA stimulation combined with anti-apoptotic actions [39]–[41]. The increase in cell number following GIPC depletion coincides with enhanced LPA_1_ activity and presumably stems from primary effects on cell survival coupled with secondary effects on cell proliferation.

GIPC was previously shown to define the signaling specificity of β-adrenergic receptor subtypes [42]. Our finding that GIPC interacts with LPA_1_ but shows much weaker or no interaction with other LPA receptor subtypes may similarly explain the differential effect of LPA_1_ and LPA_2_ on cell migration and proliferation [35]. In the case of LPA receptors, binding to PDZ domain proteins has recently been shown to influence the signaling outcomes of different LPA receptors [43]–[45]. The PDZ proteins NHERF2 and MAGI-3 were shown to couple LPA_2_ to PLC-β3, RhoA and Erk signaling [43], [44], and two additional PDZ proteins, PDZ-RhoGEF and LARG, have been shown to interact with both LPA_1_ and LPA_2_ [45]. Because the latter proteins bind to both LPA_1_ and LPA_2_, these interactions can’t explain the different effects of LPA_1_ and LPA_2_ on cell behavior [30], [35].

Shano et. al. [46] recently reported that a point mutation in the LPA_1_ PDZ binding motif led to increased Akt signaling and cell proliferation. Our findings that GIPC binds to the PDZ binding motif of LPA_1_ and depletion of GIPC has similar effects suggests that the findings of Shano et al can be explained by loss of interaction of LPA_1_ with GIPC. In contrast, loss of interaction between LPA_1_ and the PDZ proteins PDZ-RhoGEF and LARG [45] had different consequences suggesting that these proteins do not mediate the effects on Akt and cell proliferation. Previously it was shown that deletion of the LPA_1_ PDZ binding motif enhances Akt signaling [46] but did not affect inositol phosphate production [21]. These observations suggest that Akt enhancement is mediated by PLC- and inositol phosphate-independent mechanisms [13].

We previously discovered that in PC12 cells, GIPC binds to APPL on peripheral endosomes and that depletion of GIPC slows down endocytosis and trafficking of TrkA and the Rab5-effector APPL to early EEA1 endosomes [11]. Here we show that GIPC depletion similarly delays trafficking of LPA_1_ to early EEA1 endosomes and prolongs the residence of LPA_1_ receptor on APPL1 signaling endosomes. Despite the fact that GIPC depletion has similar effects on the trafficking of TrkA and LPA1, their signaling outcomes differ: GIPC depletion reduced TrkA mediated Akt and Erk signaling but enhanced LPA1 mediated Akt signaling [11]. This illustrates that signaling outcomes can be widely divergent among different receptors. Signaling depends on protein-protein interaction networks, and each receptor has a distinctive set of binding partners. TrkA and LPA1 are representatives of two diverse families, the receptor tyrosine kinases (RTKs) and G protein coupled receptors (GPCR), respectively, which have very different modes of signaling. As discussed earlier, even closely related receptors, such as LPA1, LPA2 and LPA3, form distinct protein-protein interactions with distinct signaling outcomes. Thus the molecular mechanisms underlying the different effects of GIPC depletion on TrkA and LPA1 signaling will be fully understood only when their specific binding partners and protein interaction networks are established.

Urs et al reported that deletion of the PDZ binding motif of LPA_1_ did not affect inositol phosphate signaling or the amount of LPA_1_ receptor that accumulated at the surface of HeLa cells 30 min after ligand stimulation [22]. The lack of effect on receptor accumulation suggests that the PDZ binding motif is not required for internalization of receptor from the surface. Indeed, we and others have previously shown that binding of GIPC to the PDZ motif does not promote internalization of receptors from the surface but rather promotes trafficking of receptors from peripheral signaling endosomes to early endosomes [11], [12], [17]. The association between LPA_1_ and APPL and the effects of GIPC on LPA_1_ trafficking further expand the role of GIPC and APPL to regulation of the activity of G-protein coupled receptors.

We found here that following ligand stimulation, LPA_1_ internalizes and traffics through APPL peripheral endosomes on its way to EEA1 early endosomes. Our results are in keeping with previous findings showing that ligand induced endocytosis of LPA_1_ is dependent on dynamin2 and Rab5 and that internalized LPA_1_ traverses the same endocytic pathway as the transferrin receptor in that it passes through sorting endosomes, early (EEA1) endosomes and juxtanuclear recycling endosomes [33]. GIPC is believed to affect receptor trafficking in part by binding to the Rab5 effector APPL [16], [47] and in part by binding to the actin based motor protein myosin VI [12], [48], [49]. We demonstrated that APPL associates with LPA_1_ complexes and colocalizes with LPA_1_ in peripheral endosomes. We also found that APPL depletion inhibits Akt signaling in cells expressing LPA_1_. This is in keeping with previous reports that APPL is required for activation of Akt on endosomes and for cell survival [11], [14]–[16], [50]. It appears that GIPC depletion prolongs LPA_1_ association with APPL signaling endosomes by delaying LPA_1_ trafficking to early (EEA1) endosomes, leading to increased Akt signaling and promoting cell proliferation and motility.

Our finding that interaction between GIPC and LPA_1_ leads to downregulation of Akt signaling has important pathophysiological implications, given 1) that LPA_1_ has been shown to promote the development of various carcinomas, 2) that mutations in the PDZ binding motif of LPA_1_ induces oncogenic transformation [1], [42]–[44], [46], [51], [52], and 3) that GIPC plays a tumor suppressor role in breast cancer progression [51], [53]. Whether and how the interaction of these two proteins is abrogated during cancer progression remains unknown.

In summary, the identification of signaling pathways involving GIPC and APPL downstream of LPA_1_ extend the role of these proteins as regulators of GPCRs and opens exciting directions for investigation. The ability of GIPC to bind LPA_1_, APPL and myosin VI in a ligand dependent manner positions GIPC as a key target for regulation of LPA_1_ activities. GIPC was previously shown to interact with additional GPCRs, including the dopamine D2 receptor and the lutropin receptor, but it is not known if APPL also associates with these receptors. Future studies will reveal if GIPC and APPL regulate signaling and trafficking of these and other GPCRs.

## Supporting Information

Figure S1 **Characterization of HEK-LPA_1_ cell lines stably expressing FLAG-LPA_1_.** A, Immunoblot of LPA_1_ from HEK-LPA_1_ cell lysates demonstrating receptor expression and glycosylation. A prominent broad band at ∼60 kD is seen in HEK-LPA_1_ cells (Lane 1) but not in HEK-pIRES controls stably expressing empty vector (lane 2). The electrophoretic mobility of FLAG-LPA_1_ shifts to the predicted theoretical molecular mass (38 kD) following treatment with PNGase-F (Lane 3) which removes N-glycans. The broad mobility and fuzziness of the 38 kD band most likely is due to remaining O-glycans. Lysates from HEK-LPA_1_ and HEK-pIRES cells were treated with PNGase (lanes 3–4) or sham treated (lanes 1–2), and proteins were immunoblotted with anti-FLAG IgG. B, LPA (0.01–1 µM) induces phosphorylation of Erk and Akt in HEK-LPA_1_ cells (lanes 2, 4, 6 and 8) but not in HEK-pIRES cells (lanes 1, 3, 5 and 7). HEK-LPA_1_ and HEK-pIRES cells were serum starved overnight, stimulated with the indicated amounts of LPA in 0.1% fatty acid free BSA for 5 min, lysed and analyzed by immunoblotting for LPA_1_ (FLAG), pErk, tErk, and pAkt. C, Phase contrast microscopy of HEK-pIRES and HEK-LPA_1_ cells showing that stable expression of LPA_1_ induces morphological changes in HEK293 cells. HEK-pIRES controls exhibit elongated processes (arrowheads, left panel) and overall morphology similar to the parental HEK293 cell line whereas HEK-LPA_1_ cells are flatter, more spread out and have shorter cell processes (right panel).
(TIF)

Figure S2 **FLAG-LPA_1_ and GIPC colocalize at the plasma membrane in HeLa cells.** A, Endogenous GIPC (red, in merged image) is widely distributed throughout the cytoplasm and is also concentrated along the plasma membrane whereas LPA_1_-FLAG (green) is mainly localized at the plasma membrane where it partially colocalizes with GIPC as demonstrated by yellow overlapping pixels (arrowheads, right lower panel). HeLa cells were transfected with FLAG-LPA_1_ and subsequently serum starved and processed for immunofluorescence using affinity purified rabbit anti-GIPC and mouse anti-FLAG IgG followed by goat anti-rabbit Alexa-593 and goat-anti-mouse Alexa-488 F(ab’)_2_ and examined with an Olympus FluoView 1000 confocal microscope using a 60X objective.
(TIF)

Figure S3 **LPA_1_ receptor trafficking and its colocalization with GIPC at the PM.** *Upper panel*: In serum starved cells GIPC (green) is concentrated at the plasma membrane were it colocalizes (yellow pixels, arrowheads) with LPA_1_ (red). *Lower panel* : At 30 min following stimulation, colocalization of LPA_1_ with GIPC is greatly diminished. Boxed regions are enlarged (3.2×) in the insets. HEK-LPA_1_ cells were stimulated with 10 µM LPA, processed for immunofluorescence, and images acquired exactly as for Fig. 2. Bar  = 10 µm.
(TIF)

Figure S4 **LPA_1_ traffics through APPL positive endosomes enroute to EEA1 positive early endosomes.** *Upper panel* : 2 min following stimulation with LPA (10 µM), LPA_1_ (red) colocalizes (arrowheads) with APPL (green) in endocytic vesicles at the cell periphery. 30 min following LPA stimulation, LPA_1_ appears in internal vesicles and does not colocalize with APPL. *Lower panel* : 15 and 30 min following stimulation with LPA (10 µM), LPA_1_ (red) partially colocalizes (arrowheads) with EEA1 (green). Boxed regions are enlarged (3.2×) in the insets. HEK-LPA_1_ cells were stimulated, processed for immunofluorescence and images acquired as described for Fig. 2. Bar  = 10 µm.
(TIF)

Figure S5 **LPA_1_ and GIPC are internalized into clathrin coated vesicles.** *Upper panels* : 3 min after stimulation with LPA (10 µM), LPA_1_ receptors (red) colocalize (arrowheads) with clathrin (green) on punctate structures at the plasma membrane and in endocytic vesicles immediately below the plasma membrane. *Lower panels* : GIPC (red) colocalizes (arrowheads) with clathrin (green) at the plasma membrane and on endocytic vesicles at 3 min after LPA stimulation. Boxed regions are enlarged (2.3×) in the insets. HEK-LPA_1_ cells were stimulated, processed for immunofluorescence and images acquired exactly as described for Fig. 2. Bar  = 10 µm.
(TIF)
