# Supplementary figures and images for: The PDZ Protein GIPC Regulates Trafficking of the LPA1 Receptor from APPL Signaling Endosomes and Attenuates the Cell’s Response to LPA
Source: PLoS One. 2012 Nov 8;7(11):e49227. doi: 10.1371/journal.pone.0049227 (PMC3493537; doi:10.1371/journal.pone.0049227)

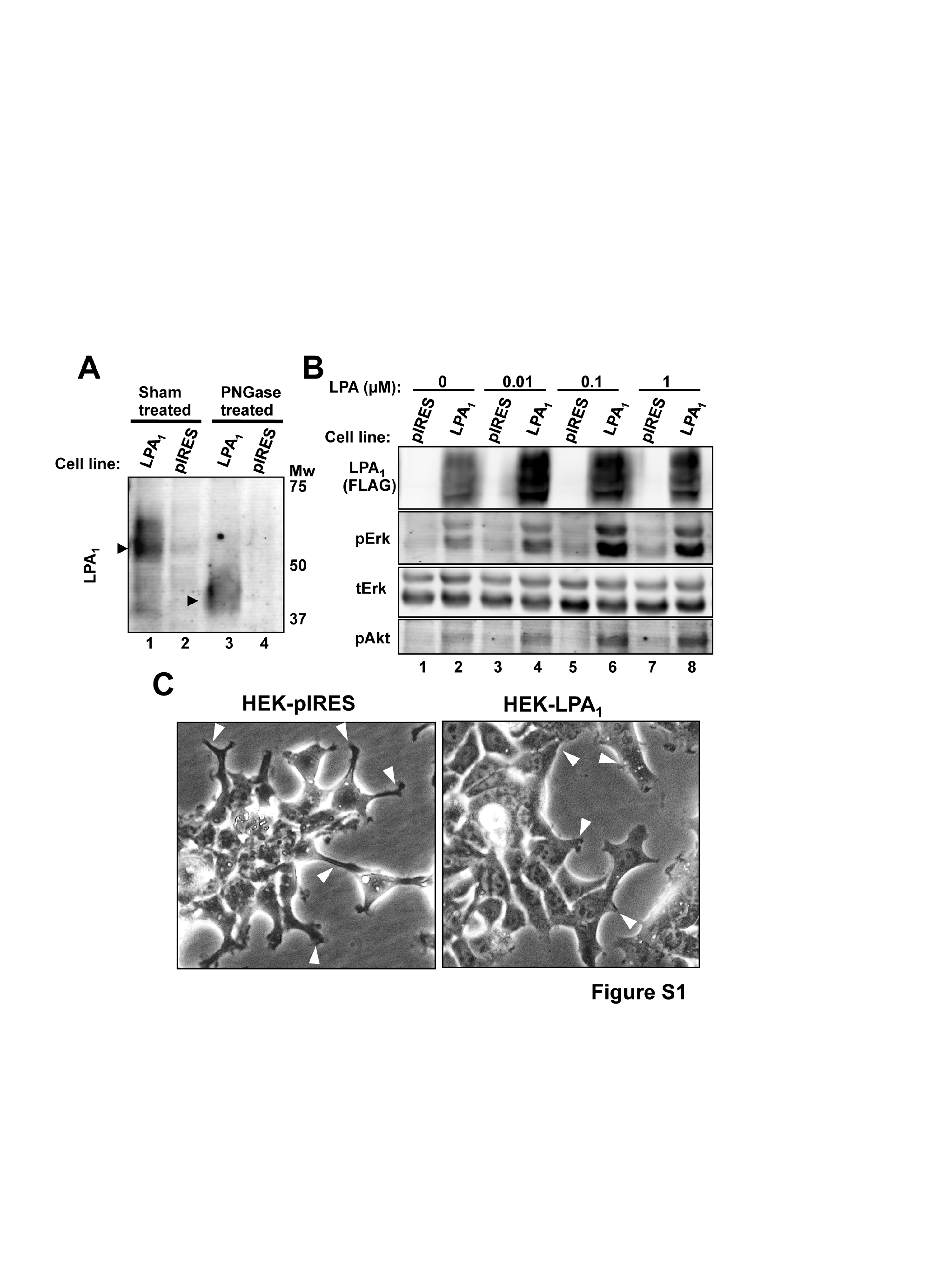

Supplement: Figure S1 — Characterization of HEK-LPA1 cell lines stably expressing FLAG-LPA1. A, Immunoblot of LPA1 from HEK-LPA1 cell lysates demonstrating receptor expression and glycosylation. A prominent broad band at ∼60 kD is seen in HEK-LPA1 cells (Lane 1) but not in HEK-pIRES controls stably expressing empty vector (lane 2). The electrophoretic mobility of FLAG-LPA1 shifts to the predicted theoretical molecular mass (38 kD) following treatment with PNGase-F (Lane 3) which removes N-glycans. The broad mobility and fuzziness of the 38 kD band most likely is due to remaining O-glycans. Lysates from HEK-LPA1 and HEK-pIRES cells were treated with PNGase (lanes 3–4) or sham treated (lanes 1–2), and proteins were immunoblotted with anti-FLAG IgG. B, LPA (0.01–1 µM) induces phosphorylation of Erk and Akt in HEK-LPA1 cells (lanes 2, 4, 6 and 8) but not in HEK-pIRES cells (lanes 1, 3, 5 and 7). HEK-LPA1 and HEK-pIRES cells were serum starved overnight, stimulated with the indicated amounts of LPA in 0.1% fatty acid free BSA for 5 min, lysed and analyzed by immunoblotting for LPA1 (FLAG), pErk, tErk, and pAkt. C, Phase contrast microscopy of HEK-pIRES and HEK-LPA1 cells showing that stable expression of LPA1 induces morphological changes in HEK293 cells. HEK-pIRES controls exhibit elongated processes (arrowheads, left panel) and overall morphology similar to the parental HEK293 cell line whereas HEK-LPA1 cells are flatter, more spread out and have shorter cell processes (right panel). (TIF) [file pone.0049227.s001.tif]

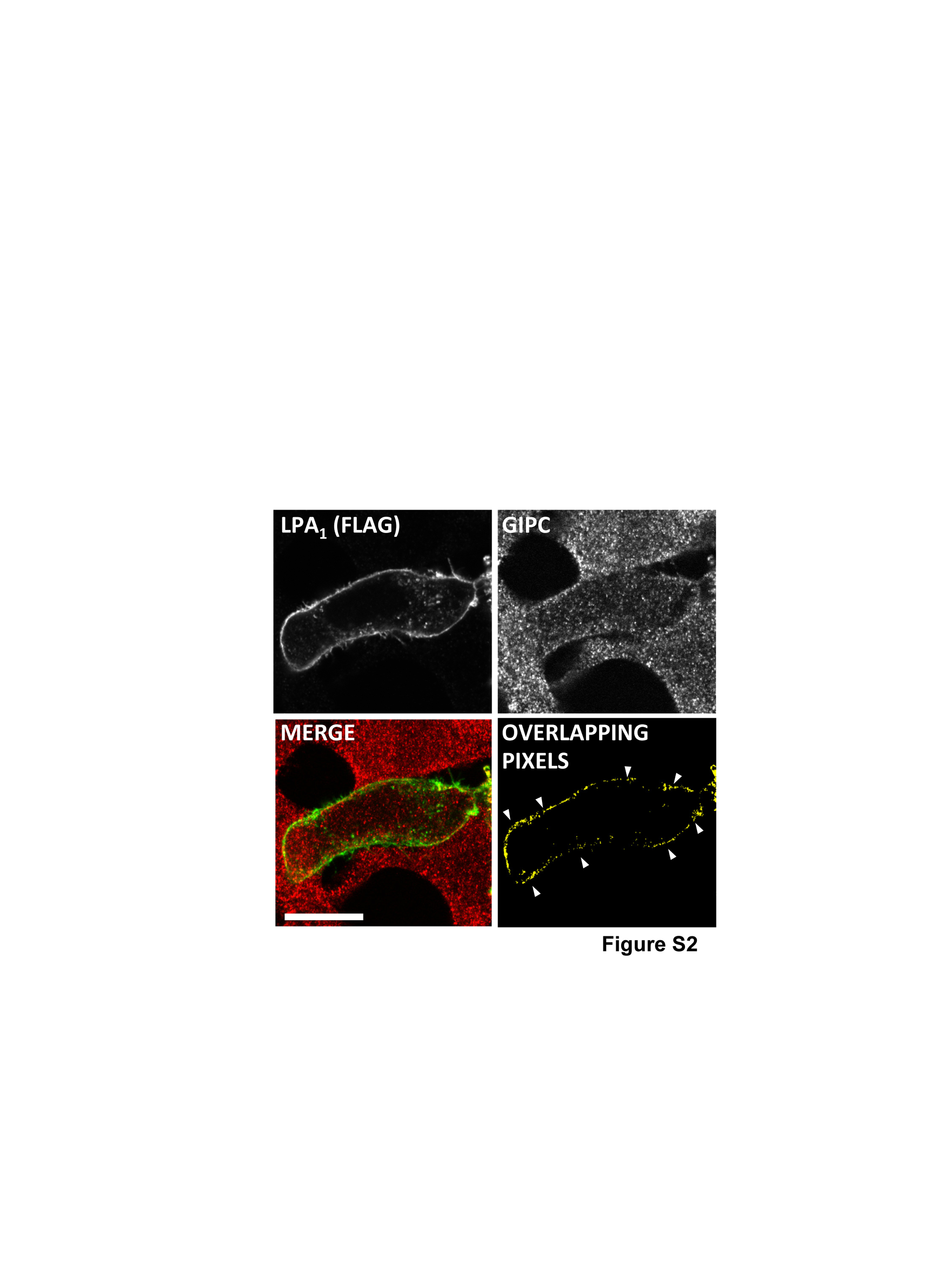

Supplement: Figure S2 — FLAG-LPA1 and GIPC colocalize at the plasma membrane in HeLa cells. A, Endogenous GIPC (red, in merged image) is widely distributed throughout the cytoplasm and is also concentrated along the plasma membrane whereas LPA1-FLAG (green) is mainly localized at the plasma membrane where it partially colocalizes with GIPC as demonstrated by yellow overlapping pixels (arrowheads, right lower panel). HeLa cells were transfected with FLAG-LPA1 and subsequently serum starved and processed for immunofluorescence using affinity purified rabbit anti-GIPC and mouse anti-FLAG IgG followed by goat anti-rabbit Alexa-593 and goat-anti-mouse Alexa-488 F(ab’)2 and examined with an Olympus FluoView 1000 confocal microscope using a 60X objective. (TIF) [file pone.0049227.s002.tif]

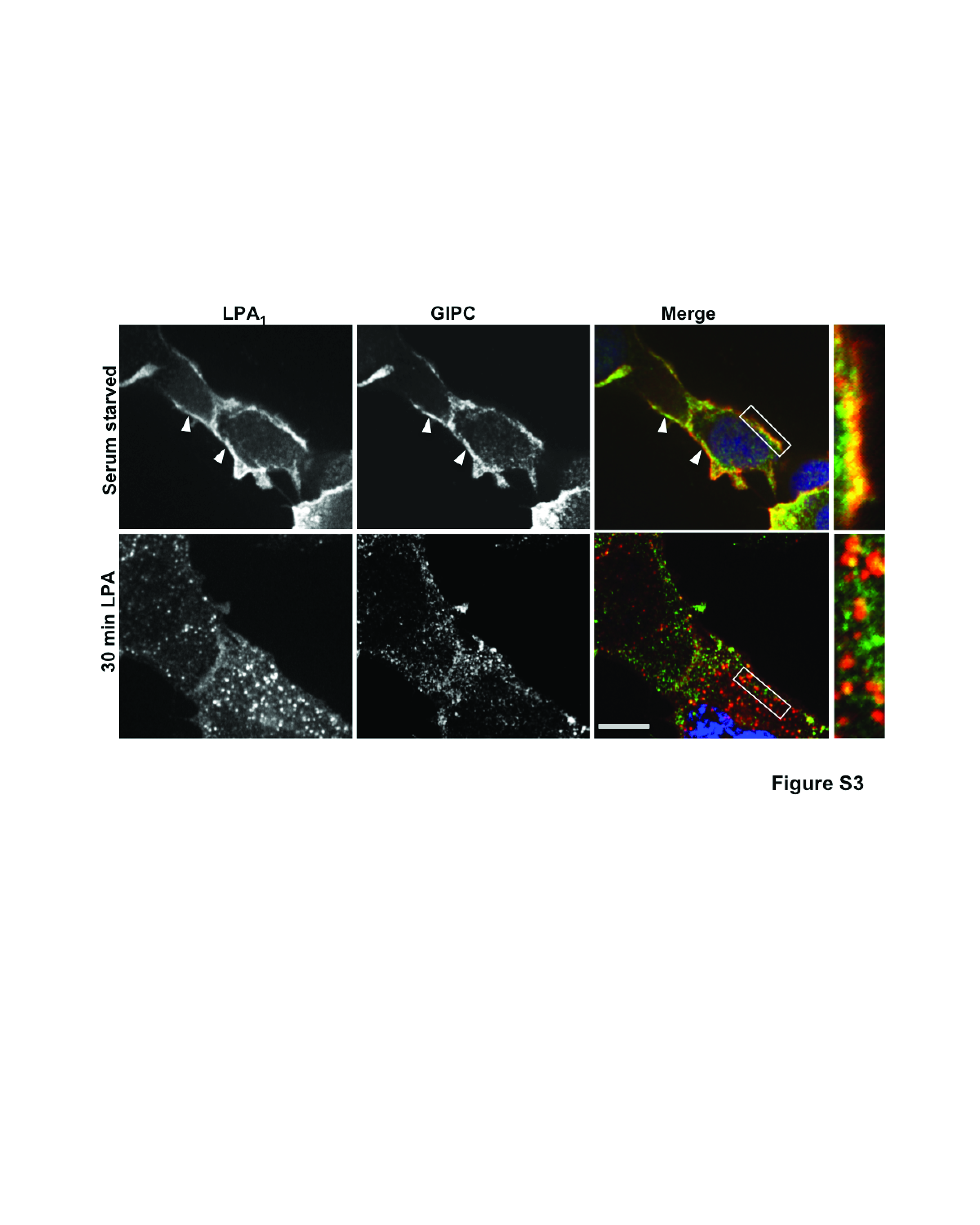

Supplement: Figure S3 — LPA1 receptor trafficking and its colocalization with GIPC at the PM. Upper panel: In serum starved cells GIPC (green) is concentrated at the plasma membrane were it colocalizes (yellow pixels, arrowheads) with LPA1 (red). Lower panel : At 30 min following stimulation, colocalization of LPA1 with GIPC is greatly diminished. Boxed regions are enlarged (3.2×) in the insets. HEK-LPA1 cells were stimulated with 10 µM LPA, processed for immunofluorescence, and images acquired exactly as for Fig. 2. Bar = 10 µm. (TIF) [file pone.0049227.s003.tif]

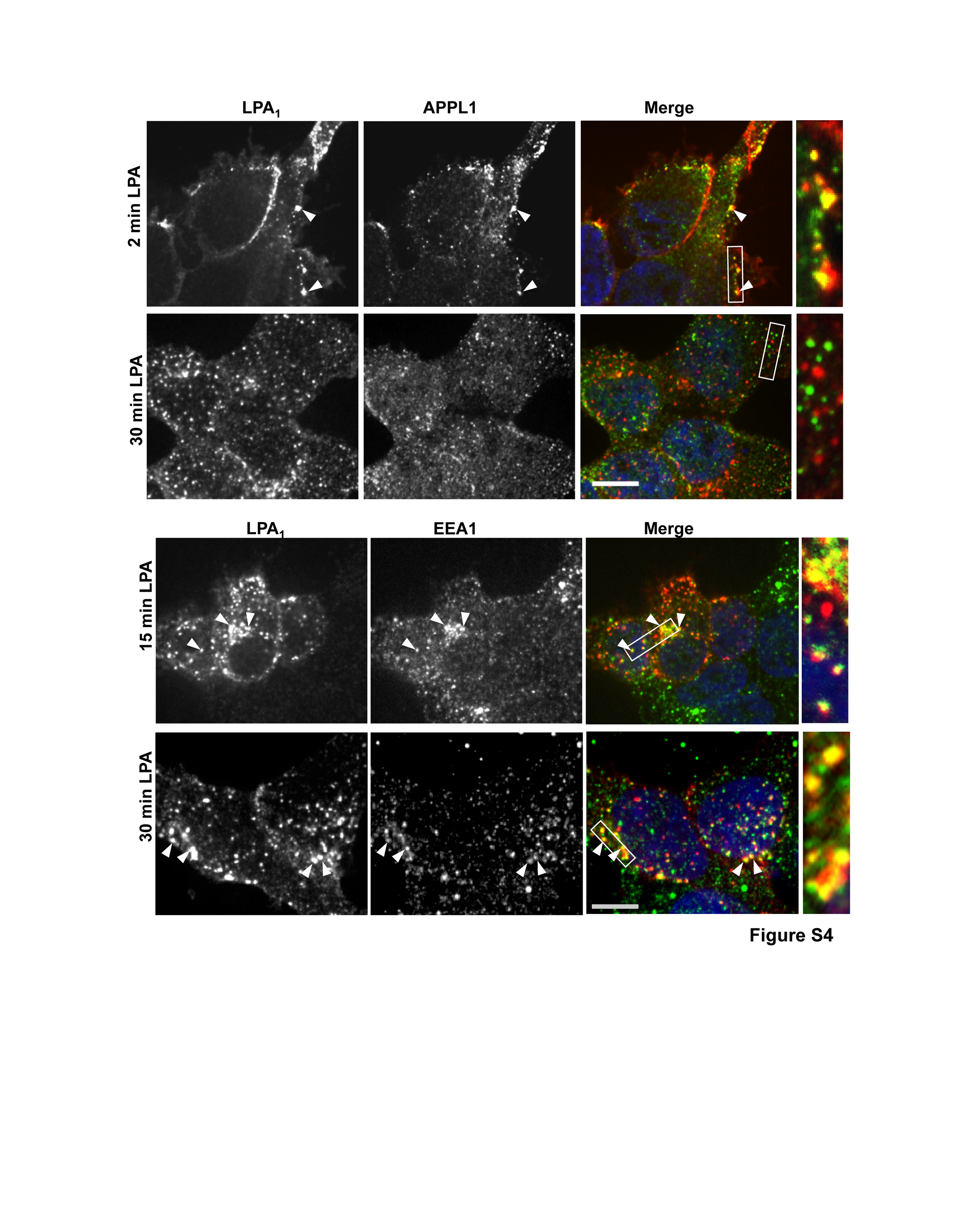

Supplement: Figure S4 — LPA1 traffics through APPL positive endosomes enroute to EEA1 positive early endosomes. Upper panel : 2 min following stimulation with LPA (10 µM), LPA1 (red) colocalizes (arrowheads) with APPL (green) in endocytic vesicles at the cell periphery. 30 min following LPA stimulation, LPA1 appears in internal vesicles and does not colocalize with APPL. Lower panel : 15 and 30 min following stimulation with LPA (10 µM), LPA1 (red) partially colocalizes (arrowheads) with EEA1 (green). Boxed regions are enlarged (3.2×) in the insets. HEK-LPA1 cells were stimulated, processed for immunofluorescence and images acquired as described for Fig. 2. Bar = 10 µm. (TIF) [file pone.0049227.s004.tif]

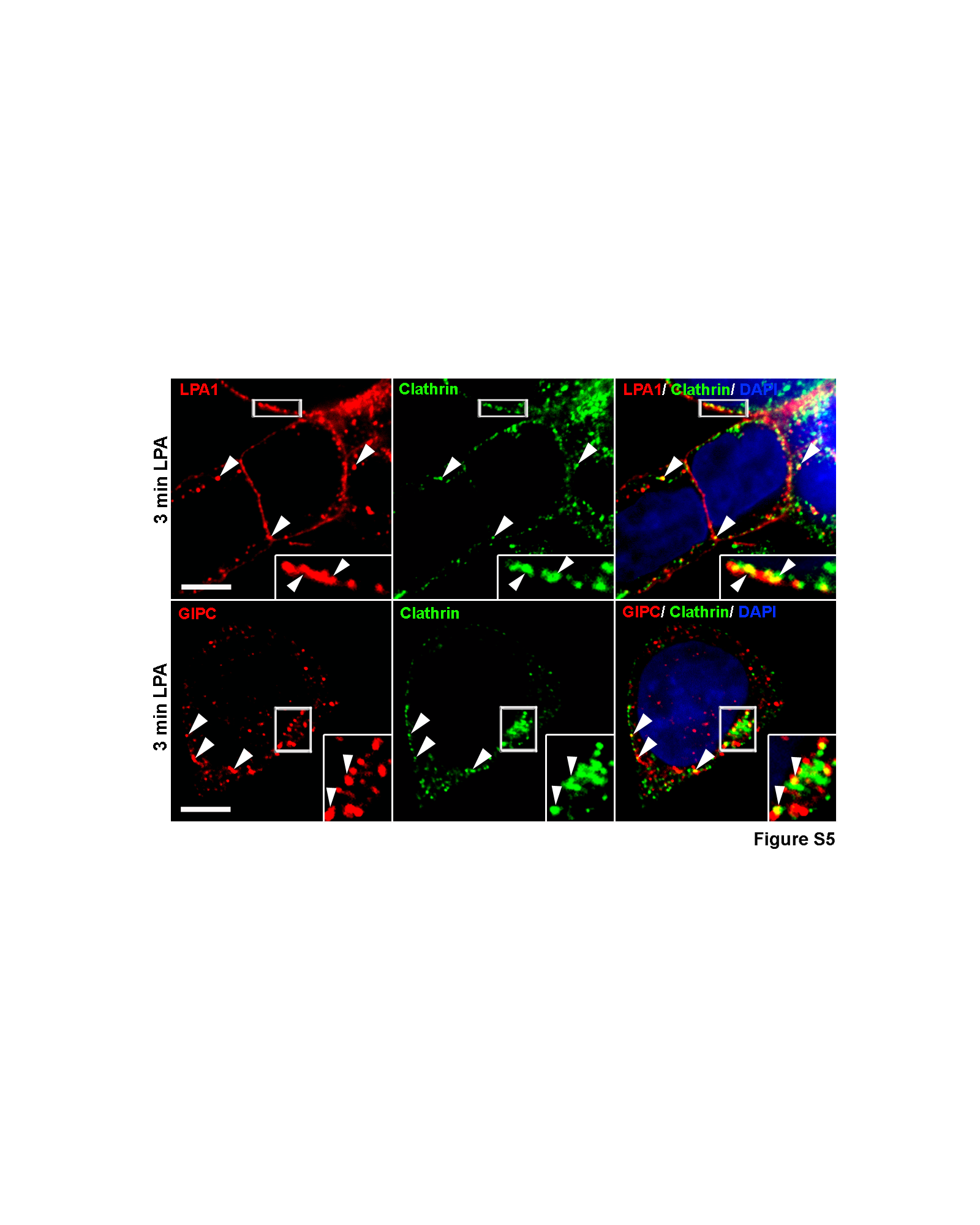

Supplement: Figure S5 — LPA1 and GIPC are internalized into clathrin coated vesicles. Upper panels : 3 min after stimulation with LPA (10 µM), LPA1 receptors (red) colocalize (arrowheads) with clathrin (green) on punctate structures at the plasma membrane and in endocytic vesicles immediately below the plasma membrane. Lower panels : GIPC (red) colocalizes (arrowheads) with clathrin (green) at the plasma membrane and on endocytic vesicles at 3 min after LPA stimulation. Boxed regions are enlarged (2.3×) in the insets. HEK-LPA1 cells were stimulated, processed for immunofluorescence and images acquired exactly as described for Fig. 2. Bar = 10 µm. (TIF) [file pone.0049227.s005.tif]
